# Antibiotic Selection for Suspected Neisseria gonorrhoeae Infection Among Penicillin-Allergic Patients in the Emergency Department

**DOI:** 10.7759/cureus.15323

**Published:** 2021-05-29

**Authors:** Matthew J McGuinness, Jonathan Mccoy, Tanaya Bhowmick

**Affiliations:** 1 Medicine, Rutgers Robert Wood Johnson Medical School, New Brunswick, USA; 2 Department of Emergency Medicine, Rutgers Robert Wood Johnson Medical School, New Brunswick, USA; 3 Department of Allergy, Immunology, and Infectious Diseases, Rutgers Robert Wood Johnson Medical School, New Brunswick, USA

**Keywords:** sti, gonorrhea, cephalosporin, penicillin, allergy, emergency department

## Abstract

Objectives

While penicillin allergies are commonly reported, their cross-reactivity with beta-lactam antibiotics is minimal. First-line treatment of gonorrheal infections includes a cephalosporin. In emergency department (ED) environments, physicians must consider these potential allergies when selecting antibiotics for a patient with symptoms concerning for sexually transmitted infection (STI).

Methods

A retrospective chart review of adult patients with symptoms concerning for STI presenting to an urban ED from January 2014 through June 2019 was performed. Chart discovery used search terms of “STI”, “STD”, “urethritis”, “vaginitis”, and “gonorrhea”. Information abstracted included patient symptoms, type of care provider, antibiotics prescribed or administered in the ED.

Results

A total of 603 patients met inclusion criteria, of which 31 reported allergies to penicillin antibiotics, and another three reported allergies to cephalosporins. Patients reporting penicillin allergy were less likely to receive a cephalosporin antibiotic (p=0.0081). Patients reporting a non-anaphylactic allergy to penicillin received a cephalosporin at a rate of 92.3%. Patients reporting a penicillin allergy under the care of only an attending physician were less likely to receive a cephalosporin antibiotic compared with those whose care teams included either a resident physician or physician assistant (p=0.00019). Patients reporting a penicillin allergy were more likely to receive alternative antibiotics beyond cephalosporins or azithromycin (p=0.048); the most frequently given additional antibiotics were metronidazole, doxycycline, and levofloxacin.

Conclusions

Patients with penicillin allergies represent a recurring challenge for ED physicians when faced with antibiotic selection for STI symptoms concerning for gonorrheal infection. Those with penicillin allergies are significantly less likely to receive a cephalosporin antibiotic, though these remain the only universally accepted treatment for gonorrheal infections. These findings highlight the significant need for further physician and public education on allergies and antibiotic selection.

## Introduction

While many patients report penicillin allergy, there is concern about the validity of these allergies. Related to this concern is the prescribing of cephalosporin antibiotics to patients with penicillin allergy. While both penicillins and cephalosporins share a beta-lactam chemical structure, evidence has shown little cross-reactivity between the molecules, implying low risk for cephalosporin allergy among those with penicillin allergy [1,2]. Nonetheless, there remains a slight risk of serious allergic reaction in patients reporting penicillin allergy who are given cephalosporins [2]. These decisions are further complicated by the continued rise of antimicrobial resistance [1].

Emergency departments (ED) remain one area where clinical decisions about allergies must occur without time for allergy testing [3]. Sexually transmitted infections (STIs) represent a large number of ED visits annually, with some speculation that gonorrhea and chlamydia infections are under-recognized in the ED [4-6]. ED visits related to STI complaints continue to grow in the United States, with a 38.6% rise in STI-related ED visits from 2008-2010 to 2011-2013 [7].

The current Center for Disease Control (CDC) guidelines recommend the use of ceftriaxone in the treatment of gonorrhea infection, raising potential concern among patients with these infections who also report a penicillin allergy [8]. Of note, there is no cross-reactivity or concern for these patients with regard to co-treatment for Chlamydia trachomatis. Further complicating this issue is the lack of consensus on a treatment guideline for gonorrhea patients with confirmed penicillin or cephalosporin allergy [8-10]. The CDC suggests a possible alternative treatment with combined gentamicin and azithromycin for patients with known cephalosporin allergy, but there remains little study of this regimen, and no updated guidance from the Infectious Diseases Society of America at the time of this publication [8]. In determining their choice of antibiotic, ED physicians will not have follow-up appointments with patients, and so cannot track resolution of infection. Without clear recommendations for approaching STI treatment in penicillin-allergic patients, ED physicians may be forced to make more subjective decisions about patient care.

This study examined the frequency of reported penicillin allergy among patients presenting to the ED with symptoms of gonorrhea. Furthermore, this study assessed if reported the penicillin allergy impacted treatment decisions of ED physicians. Ascertaining the scope of this problem will encourage further investigation into the treatment of ED patients with penicillin allergy presenting for conditions for which penicillin or cephalosporins are recommended. This study was presented as a poster at the Open Forum Infectious Disease, 2020.

## Materials and methods

A retrospective chart review was conducted on patients with symptoms concerning for sexually transmitted infection evaluated at an urban ED with approximately 80,000 annual visits. All patients over the age of 21 who were evaluated for, diagnosed with, or treated for gonorrhea infection were included. Patients aged 18-21 were excluded due to possible treatment within a designated Pediatric ED. The academic IRB review board approved this study’s protocol.

Chart discovery was performed using Vizient Clinical Data Base-Resource Manager (CDB/RM^TM^, Vizient Inc., Irving, TX), which contains discharge abstract data on all patient visits to the ED. The data extracted includes demographic information and diagnosis codes for specific patient encounters [11]. Patient charts were analyzed between January 2014 and June 2019 and were screened using search terminology including “STI”, “STD”, “urethritis”, “vaginitis”, and “gonorrhea”. Data abstraction sheets were developed to record demographics and objective information about each patient visit, including International Classification of Diseases (ICD) diagnosis codes, reported allergies, antibiotic prescriptions and administration of antibiotics, and the role of the provider who most closely worked with the patient (resident physician, physician assistant, or attending physician).

The primary outcome for this study focused on the prevalence of reported allergy to penicillins and cephalosporins among patients in the ED reporting STI symptoms. Secondary outcomes examined the rate of certain antibiotic treatments in the care of patients with these allergies compared to those without.

Descriptive statistics were used to evaluate demographic factors and clinical variables. Analysis was performed on antibiotic prescriptions between patients reporting allergies to penicillins or cephalosporins, and those who did not. These comparisons were performed using chi-square tests of independence and Fischer’s exact tests. Data from the Vizient Clinical Data Base/Resource Manager^TM^ was used with permission from Vizient, all rights reserved.

## Results

Following the chart review, 603 patients met inclusion criteria for presentation to the ED with symptoms concerning for STI and gonorrheal infection; 34 of 603 (5.6%) of patients reported an allergy to either penicillin or cephalosporins, with 31 of 603 reporting an allergy to penicillins. There was no significant difference in mean age between groups (Allergy - 31.4, No Allergy - 30.9).

While patients received antibiotics at a similar rate (Allergy - 96.8% vs. No Allergy - 91.7%), patients who reported a penicillin allergy were less likely to be prescribed a cephalosporin (Allergy - 67.7% vs. No Allergy - 85.4%, p=0.0081) (Table 1). In addition, patients reporting a penicillin allergy were more likely to receive antibiotics beyond a cephalosporin or azithromycin (Allergy - 48.4% vs. No Allergy - 30.8%, p=0.048). Though sample sizes were small, patients reporting penicillin allergies had a statistically higher likelihood to receive azithromycin without a cephalosporin (p<0.00001), and were more likely to receive azithromycin alone with no other antibiotic therapy (p=0.0023). The most frequently prescribed other antibiotics among patients reporting allergy were metronidazole (7), doxycycline (4), and levofloxacin (2). Gentamicin was prescribed to one penicillin-allergic patient who reported a non-anaphylactic allergy.

**Table 1:** Antibiotic Prescriptions for Patients with Penicillin Allergy vs. without Allergy

	Penicillin Allergy Reported (n=31)	No Allergy Reported (n=569)	p-value
Age (years), mean (range)	31.4 (22-59)	30.9 (22-66)	
Male sex, no. (%)	28 (90.3)	489 (85.9)	
Any Antibiotics given (%)	30 (96.8)	522 (91.7)	
Cephalosporin given (%)	21 (67.7)	486 (85.4)	p = 0.0081
Azithromycin given (%)	26 (83.9)	450 (79.1)	
Any other antibiotic given (%)	15 (48.4)	175 (30.8)	p = 0.048
# Tested for G/C (%)	30 (80.6)	474 (83.3)	
Azithromycin given without a cephalosporin (%)	6 (19.4)	3 (0.527)	p <0.00001
Azithromycin ONLY (%)	3 (9.68)	3 (0.527)	p = 0.0023
Azithromycin given WITH a non-cephalosporin antibiotic (%)	3 (9.68)	0	p =0.0001
# Patients returning to ED within 1 month for same symptoms	3 (9.68)	50 (8.79)	

Among patients reporting penicillin allergy, those reporting an “unknown” response to penicillin accounted for 16 of 31 patients. Patients claiming an “unknown” response to penicillins were less likely to receive a cephalosporin as opposed to those with a known non-anaphylactic response (Unknown - 56.3% vs. Non-Anaphylactic - 92.3%, p=0.0443). Of three patients reporting cephalosporin allergy, one patient, with known non-anaphylactic response, received a cephalosporin, while the other two did not. This patient’s reported allergy included a description of a “hives” reaction at an injection site, which their care providers likely felt was unlikely to be a true allergy.

In examining care provider decision making, patients reporting penicillin allergy who were cared for most closely by a resident physician were more likely to receive antibiotics that were neither azithromycin nor a cephalosporin, at a rate of 85.7%, compared with those cared for primarily by a physician assistant (25.0%) or attending physician (47.4%) (p=0.0033). In addition, patients with penicillin allergy cared for primarily by an attending physician were less likely to receive a cephalosporin antibiotic, at a rate of 52.6%, compared to resident physicians (71.4%) and physician assistants (87.5%) (p=0.00019).

## Discussion

Rates of STI complaints in the ED are growing [7]. Despite this, there remains no consensus guideline on treating gonorrheal infections in patients who cannot receive cephalosporin antibiotics due to allergy [8-10]. While new, the CDC has issued new recommendations on a regimen for addressing this issue; there remains little study of the regimen and it is not advised for pharyngeal gonorrhea, leaving no alternate regimen to consider for management of pharyngeal infection [8]. This study examined the rate of penicillin and cephalosporin allergy among these patients to assess the burden of this problem on ED providers. About 5.6% of patients reported allergy to penicillin or cephalosporin, representing a sizable proportion of patients with STI complaints needing treatment. Despite reported cross-reactivity with cephalosporins as low as 1%, this study demonstrates concern among ED care providers when it comes to prescribing cephalosporins for penicillin-allergic patients with STI symptoms [3].

Concern about cephalosporin prescribing practices is most relevant to gonorrheal infections, given the lack of fully validated alternative regimens [8,9]. Regardless, ED care providers appear to provide non-cephalosporin antibiotics to those with penicillin allergies at a rate higher than that of those without allergies. This finding was most notable with attending physicians, who prescribed cephalosporins less often than when the care team involved a resident physician or physician assistant. This raises unease in considering that patients could go undertreated, or potentially contribute to rising fears of antimicrobial resistance.

This study has several limitations. First, this is a single-center, retrospective study, which somewhat limits generalizability, and may not fully reflect the current landscape of antibiotic prescribing practices in EDs. In addition, the retrospective nature of this trial confines the amount of information for patient encounters to what is included in the medical record. Discussions of patient symptoms or allergy severity which were not documented in the medical record, or were otherwise not clearly outlined, may have been missed in retrospective review. Similarly, a select number of search terms were utilized to find patient records meeting inclusion criteria. These search terms were by no means exhaustive and therefore, could mean certain patient records may have been missed. Finally, the inclusion criteria utilized excluded patients between 18-21 years of age as they may be treated as pediatric patients. This is an important limitation, as young patients comprise a large portion of those presenting with STI complaints, and an unknown portion may have been treated in the pediatric ED.

## Conclusions

This study’s findings highlight the need for further education into areas surrounding allergy and antibiotic selection. The finding that attending physicians appear least likely to use a cephalosporin for patients reporting penicillin allergy could imply that prior education did not focus specifically on antibiotic allergies in which non-anaphylactic reactions could allow for the use of non-penicillin beta-lactam antibiotics safely. This may highlight a need for emphasis on these areas in continuing medical education. While limited by its single-center and retrospective approaches, this study highlights the key role allergies play in antibiotic decision making, which impacts care providers in numerous settings. Future endeavors should focus on the education of care providers, and encourage care providers to work with colleagues from specialized fields including infectious diseases, allergy, and immunology in making decisions about antibiotic prescriptions in allergic patients. Finally, improved decision-making tools and patient resources will be essential in ED settings to ensure that the growing number of ED patients are treated effectively.
